# ^1^H-NMR-based metabolic profiling identifies non-invasive diagnostic and predictive urinary fingerprints in 5q spinal muscular atrophy

**DOI:** 10.1186/s13023-021-02075-x

**Published:** 2021-10-20

**Authors:** Afshin Saffari, Claire Cannet, Astrid Blaschek, Andreas Hahn, Georg F. Hoffmann, Jessika Johannsen, Romy Kirsten, Musa Kockaya, Stefan Kölker, Wolfgang Müller-Felber, Andreas Roos, Hartmut Schäfer, Ulrike Schara, Manfred Spraul, Friedrich K. Trefz, Katharina Vill, Wolfgang Wick, Markus Weiler, Jürgen G. Okun, Andreas Ziegler

**Affiliations:** 1 Division of Child Neurology and Metabolic Medicine, Center for Child and Adolescent Medicine, Heidelberg University Hospital, Im Neuenheimer Feld 430, 69120 Heidelberg, Germany; 2 Bruker BioSpin GmbH, Rheinstetten, Germany; 3 Division of Pediatric Neurology and Developmental Medicine and LMU Center for Children With Medical Complexity, LMU Hospital, Dr. von Hauner Children’s Hospital, Munich, Germany; 4 Department of Child Neurology, University Hospital Gießen, Gießen, Germany; 5 Department of Pediatrics, Neuropediatrics, University Medical Center Hamburg-Eppendorf, Hamburg, Germany; 6 NCT Liquidbank, National Center for Tumor Diseases, Heidelberg, Germany; 7 Buchener Str. 12, Mannheim, Germany; 8 Department of Neuropediatrics, Developmental Neurology and Social Pediatrics, Centre for Neuromuscular Disorders in Children, Children’s University Clinic Essen, University of Duisburg-Essen, Essen, Germany; 9 Department of Neurology, Heidelberg University Hospital, Heidelberg, Germany

**Keywords:** Spinal muscular atrophy, SMA, Metabolic profiling, ^1^H-nuclear magnetic resonance, NMR, Urinary fingerprints

## Abstract

**Background:**

5q spinal muscular atrophy (SMA) is a disabling and life-limiting neuromuscular disease. In recent years, novel therapies have shown to improve clinical outcomes. Yet, the absence of reliable biomarkers renders clinical assessment and prognosis of possibly already affected newborns with a positive newborn screening result for SMA imprecise and difficult. Therapeutic decisions and stratification of individualized therapies remain challenging, especially in symptomatic children. The aim of this proof-of-concept and feasibility study was to explore the value of ^1^H-nuclear magnetic resonance (NMR)-based metabolic profiling in identifying non-invasive diagnostic and prognostic urinary fingerprints in children and adolescents with SMA.

**Results:**

Urine samples were collected from 29 treatment-naïve SMA patients (5 pre-symptomatic, 9 SMA 1, 8 SMA 2, 7 SMA 3), 18 patients with Duchenne muscular dystrophy (DMD) and 444 healthy controls. Using machine-learning algorithms, we propose a set of prediction models built on urinary fingerprints that showed potential diagnostic value in discriminating SMA patients from controls and DMD, as well as predictive properties in separating between SMA types, allowing predictions about phenotypic severity. Interestingly, preliminary results of the prediction models suggest additional value in determining biochemical onset of disease in pre-symptomatic infants with SMA identified by genetic newborn screening and furthermore as potential therapeutic monitoring tool.

**Conclusions:**

This study provides preliminary evidence for the use of ^1^H-NMR-based urinary metabolic profiling as diagnostic and prognostic biomarker in spinal muscular atrophy.

**Supplementary Information:**

The online version contains supplementary material available at 10.1186/s13023-021-02075-x.

## Background

5q spinal muscular atrophy (SMA) is an autosomal-recessive neuromuscular disease caused by homozygous deletions or loss-of-function mutations in the *survival motor neuron 1 gene (SMN1)* with retained function of at least one copy of the paralogous *SMN2* gene, resulting in a progressive loss of alpha motor neurons in the spinal cord and lower brain stem. Based on age at onset and phenotypic severity, SMA has been divided into four clinical subtypes [1, 2]: SMA 1 (“non-sitters”, onset 0–6 months), SMA 2 (“sitters but non-walkers”, onset 7–18 months), SMA 3 (“walkers”, onset before (3a) or after (3b) 3 years), and SMA 4 (adult onset).

Over the past years, novel treatments, such as the *SMN2* splicing modifiers Nusinersen [3, 4] and Risdiplam ([5, 6]; *ClinicalTrials.gov*: NCT02913482, NCT02908685), as well as Onasemnogene Abeparvovec [7], as first gene replacement therapy, have been developed for SMA. These therapies hold the potential to significantly improve disease trajectories if administered early in the disease course, preferably before first symptoms arise [8–11]. To allow early treatment initiation by pre-symptomatic diagnosis, the feasibility and medico-economic impacts of different newborn screening programs for SMA are currently being evaluated in clinical trials [12, 13] (*ClinicalTrials.gov*: NCT03655223, NCT03217578). Yet, not all patients identified by newborn screening will develop clinical signs shortly after birth and many SMA patients show an attenuated disease course with late onset of disease and only minimal muscle weakness. Due to the absence of predictive biomarkers, treatment decisions are currently based on quantification of *SMN2* copy numbers, with moderate genotype–phenotype correlation [14, 15]. The inability of reliably predicting disease severity may result in severely affected SMA patients (e.g. with 4 *SMN2* copies) being denied pre-emptive treatment and at the same time mildly affected patients being subject to costly and invasive therapies, many years before first symptoms arise. Hence, the need for reliable biomarkers is pressing [15]. Interestingly, urinary metabolic profiling has successfully been used to identify potential biomarkers in a number of central nervous system (CNS) diseases, including neuropsychiatric, demyelinating, metabolic, neurodegenerative, mitochondrial and motor neuron diseases [16–23]. Only recently, a first attempt has been made to use ^1^H-nuclear magnetic resonance (NMR)-based metabolomics as biomarker in SMA reporting an overall reduction of metabolites compared to healthy controls without evidence for metabolomic changes under Nusinersen therapy [24].

The aim of this proof-of-concept study was to use a highly standardized and strictly quality controlled NMR-based metabolomics platform to further explore whether urinary metabolic signatures can serve as additional biomarkers for diagnosis and disease prediction in SMA.

## Results

### Clinical and demographic characteristics

A total of 29 treatment-naïve SMA patients (5 pre-symptomatic, 9 SMA 1, 8 SMA 2, 7 SMA 3) were included in the study. Clinical characteristics are summarized in Table 1.

**Table 1:** Clinical characteristics of SMA patients

ID	Age	Gender	SMA type	*SMN1* mutation	*SMN2* copies	Ambula-tory	CHOP-INTEND (max. 64)	HFSME (max. 66)	RULM (max. 37)
SMA001	2 m	M	PRE	Hom. Δ7/8	4	No	59		–
SMA002	4 m	M	PRE	Hom. Δ7/8	4	No	54	–	–
SMA003	7 m	F	PRE	Hom. Δ7/8	5	No	64	–	–
SMA004	1y 2 m	F	PRE	Hom. Δ7/8	5	No	–	–	–
SMA005	3 m	F	PRE	Hom. Δ7, Het. Δ8	4	No	62	–	–
SMA006	2 m	F	1	Hom. Δ7/8	2	No	22	–	–
SMA007	4 m	F	1	Hom. Δ7/8	2	No	22	–	–
SMA008	3 m	M	1	Hom. Δ7/8	2	No	13	–	–
SMA009	5 m	M	1	Het. Δ7/8, (c.46delG)	2	No	20	–	–
SMA010	5 m	M	1	Hom. Δ7	2	No	28	–	–
SMA011	9 m	M	1	Hom. Δ7/8	3	No	32	–	–
SMA012	14y	F	1	Hom. Δ7/8	2	No	18	–	1
SMA013	16y 10 m	F	1	Hom. Δ7/8	3	No	10	–	5
SMA014	5y 5 m	F	1	Hom. Δ7	3	No	–	–	–
SMA015	1y 2 m	F	2	Hom. Δ7	3	No	–	10	–
SMA016	1y 5 m	M	2	Hom. Δ7/8	3	No	44	–	–
SMA017	2y 6 m	F	2	Hom. Δ7/8	3	No	59	12	–
SMA018	9y	M	2	Hom. Δ7/8	3	No	26	–	6
SMA019	14y 8 m	M	2	Hom. Δ7	3	No	–	7	21
SMA020	17y 8 m	F	2	Hom. Δ7/8	3	No	–	17	25
SMA021	18y	M	2	Hom. Δ7/8	3	No	–	0	6
SMA022	18y 11 m	F	2	Hom. Δ7/8	3	No	–	2	15
SMA023	3y 2 m	F	3a	Hom. Δ7/8	3	Yes	–	36	11
SMA024	8y 3 m	F	3a	Hom. Δ7/8	3	No	–	–	–
SMA025	8y 9 m	F	3a	Hom. Δ7/8	3	No	–	22	21
SMA026	12y 8 m	F	3a	Hom. Δ7/8	3	No	–	31	23
SMA027	3y 5 m	M	3b	Hom. Δ7, Het. Δ8	2	Yes	–	50	20
SMA028	4y 4 m	M	3b	Hom. Δ7/8	4	Yes	–	44	21
SMA029	14y 1 m	F	3b	Hom. Δ7/8	4	Yes	–	54	33

CHOP-INTEND, Children’s Hospital of Philadelphia Infant Test of Neuromuscular Disorders; Δ/del, deletion; F, female; het, heterozygous; HFSME, Hammersmith Functional Motor Scale Expanded; hom, homozygous; M, male; m, months; PRE, pre-symptomatic; RULM, Revised Upper Limb Module; SMA, spinal muscular atrophy; y, years

Age significantly differed among our cohort (Kruskal–Wallis Anova test, *P* = 0.0069) with median ages of 0.4 (IQR = 0.48) years for pre-symptomatic, 0.5 (IQR = 7.3) years for SMA 1, 11.9 (IQR = 15.8) years for SMA 2 and 8.3 (IQR = 8.0) years for SMA 3 patients. Gender was equally distributed with male-to-female ratios of 0.7:1 in the pre-symptomatic, 0.8:1 in the SMA 1 and 1:1 in the SMA 2 cohort. The SMA 3 cohort showed a male-to-female ratio of 0.4:1. Four patients were clinically diagnosed as SMA 3a and three as SMA 3b, all SMA 3b and one SMA 3a patient (SMA023) were ambulatory at time of sample collection. The individual *SMN1* mutations and *SMN2* copy numbers are depicted in Table 1. For disease prediction of pre-symptomatic SMA patients only those with 4 *SMN2* copies were included in the study. Longitudinal collection of therapy-naïve pre-symptomatic children with 2-3 *SMN2* copies was not feasible, since in Europe, these patients were treated with Nusinersen rapidly after birth. For pre-symptomatic individuals with SMA and 4 *SMN2* copies, treatment guidelines in Europe during the recruitment period of the study recommended a watchful waiting strategy, allowing longitudinal follow-up and correlation of predicted phenotypes according to metabolic signatures with the onset of symptoms and natural history of the disease before start of specific therapy.

Median HFSME scores (max. 66) significantly differed between SMA 2 (median 8.5, IQR = 8.3) and SMA 3 (median 40.0, IQR = 16.3) (Kruskal–Wallis Anova test, *P* = 0.0062), pleading for relevant differences in the execution of complicated motor tasks and lower limb function. Similarly, median RULM scores (max. 37), reflecting upper limb function, were significantly different (Kruskal–Wallis Anova test, *P* = 0.0493) (SMA2: 15.0 (IQR = 15.0); SMA 3:21.0 (IQR = 2.3)). Median CHOP-INTEND scores (max. 64) with 60.5 (IQR = 4.8) for pre-symptomatic, 21.0 (IQR = 6.8) for SMA 1 and 44.0 (IQR = 16.5) for SMA 2 showed differences, however, did not reach significance level (Kruskal–Wallis Anova test, *P* = 0.0502).

**Fig. 1:**
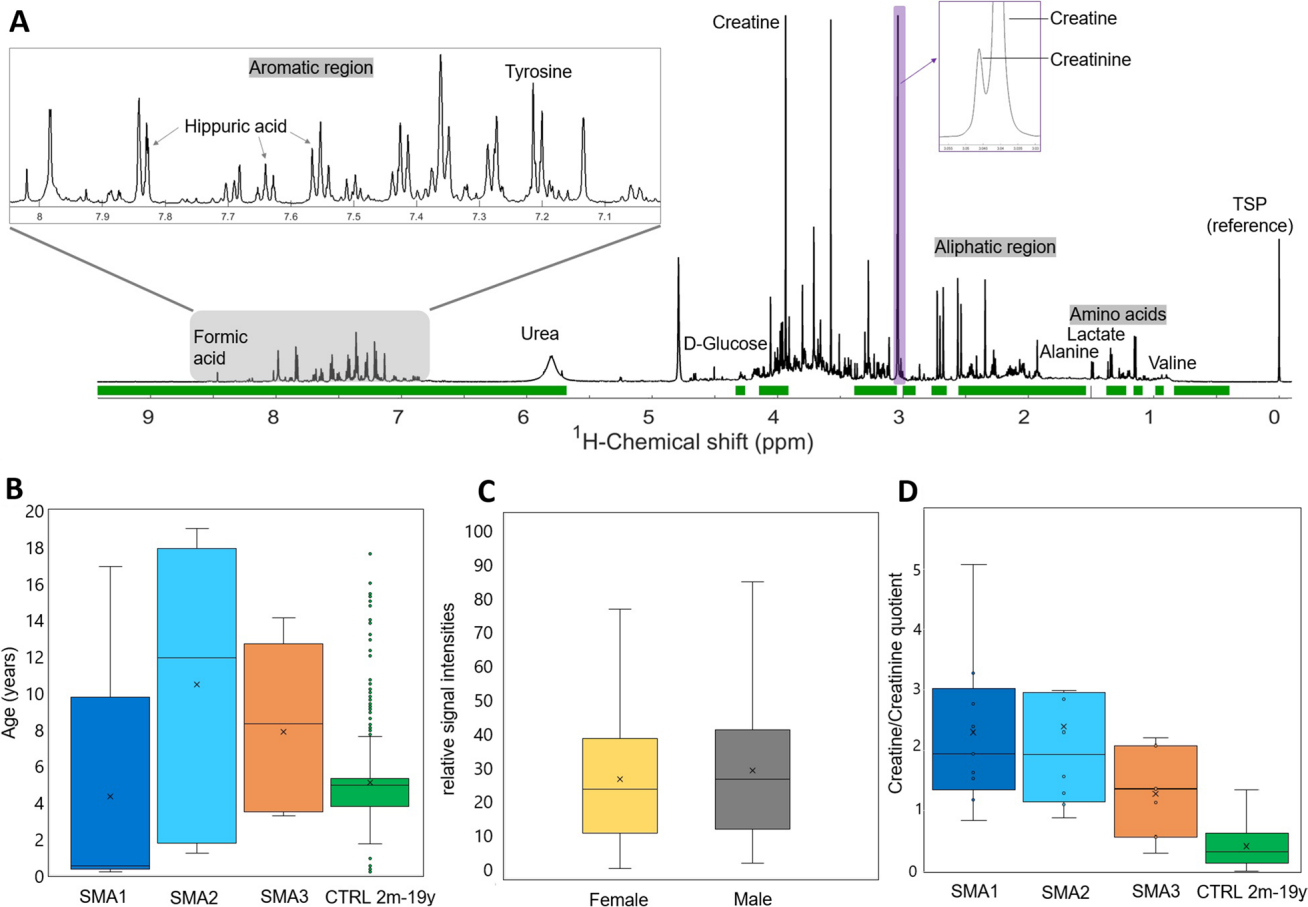
Urinary ^1^H-NMR metabolic spectrum and possible confounders. **A** Representation of the complete urinary ^1^H-NMR metabolic spectrum of a healthy individual. Exemplary metabolites and close-ups of the aromatic region as well as the creatine and creatinine peaks are shown for illustrative purposes. TSP (trimethylsilylpropionic acid-d_4_ sodium salt) served as reference peak. The spectral regions highlighted in green were used for further analysis. **B** Plotting of different SMA subgroups vs. age-matched controls revealed age as a significant confounder emphasizing the importance of age-matched experimental setups. **C** No differences were observed concerning gender. **D** Quantification of the creatine-to-creatinine quotient showed significant differences between SMA subgroups and controls. To avoid variability due to creatinine metabolism, which was recently shown to be impaired in SMA [25], the spectral regions for creatine and creatinine were excluded prior to postprocessing in the following experiments. *SMA:* spinal muscular atrophy. *CTRL:* control

A representative complete urinary ^1^H-NMR metabolic spectrum of a healthy individual is shown in Fig. 1A. As expected, age significantly differed between subgroups (*P* < 0.01) and proved to be an important confounder (Fig. 1B and Additional file 1: Fig. S1). Age bias was reduced by conducting experiments with age-matched subgroups as far as possible. No significant differences were observed concerning gender (Fig. 1C). Quantification of the urine creatine-to-creatinine quotient showed significant differences between SMA subgroups and controls (Fig. 1D), in line with recent reports [25]. However, since creatine/creatinine is foremost a marker of muscle activity/immobility and therefore highly unspecific, the ^1^H-NMR spectral regions for creatine and creatinine were excluded from analysis.

Reference urinary metabolic profiles of healthy individuals were established by a large control group of 444 healthy individuals ranging from 2 months to 19 years. Median age was 4.9 (IQR = 1.6) years, with a male-to-female ratio of 1:0.7.

### Urinary metabolic profiles can provide recognizable fingerprints for SMA

In order to identify non-invasive diagnostic fingerprints, we used an ^1^H-NMR-based metabolomics approach to analyze urinary samples of 24 SMA patients (9 SMA 1, 8 SMA 2 and 7 SMA 3) between 2 months and 19 years compared to an age-matched control group. The accuracy of the predictions for individual patients is depicted in Table 2.

**Table 2:** PCA/CA-embedded MCCV classification results for the different models

ID	SMA type	Correct classification			Comments
		SMA vs. CTRL (%)	SMA 1 vs. SMA 2 vs. SMA 3 vs. CTRL	SMA 1/2 vs. SMA 3 vs. CTRL	
SMA006	1	100	59% (*)	38% (*)	
SMA007	1	100	100%	41% (*)	
SMA008	1	4	95%	99%	
SMA009	1	100	80% (*)	92% (*)	
SMA010	1	100	100%	15% (*)	
SMA011	1	100	11% (*)	85% (*)	Mild disease course, borderline SMA 2
SMA012	1	100	58% (41% SMA 2)	100%	
SMA013	1	100	2% (98% SMA 2)	100%	Mild disease course, 3 *SMN2* copies, no respiratory involvement, possible age bias
SMA014	1	100	90% (*)	99%	
SMA015	2	0	0% (*)	0% (*)	Mild disease course, borderline SMA 3, good response to Nusinersen (standing and first steps) as potential sign for residual motor capacity
SMA016	2	100	0% (79% SMA 1)	53% (*)	
SMA017	2	100	78%	97%	
SMA018	2	100	0% (95% SMA 3)	16% (83% SMA 3)	Severe disease course, mild intellectual disability Multiple comorbidities: pulmonary hypertension due to Ebstein anomaly, epileptic seizures Medication: sildenafil, valproic acid
SMA019	2	100	28% (46% SMA 1)	96%	Severe disease course with orthopedic involvement: severe neuromyopathic scoliosis, spondylodesis
SMA020	2	34	0% (45% SMA 1)	100%	Orthopedic involvement: severe neuromyopathic scoliosis, spondylodesis
SMA021	2	100	15% (83% SMA 1)	100%	*Respiratory involvement*: s/p recurrent pneumonias, s/p pleural empyema requiring surgical intervention *Orthopedic involvement*: severe neuromyopathic scoliosis, spondylodesis with recurrent bacterial infections requiring 11 surgical revisions resulting in extensive scarring of the back
SMA022	2	100	92%	100%	
SMA023	3a	100	96%	63% (32% CTRL)	Mild disease course
SMA024	3a	100	0% (49% CTRL)	0% (83% SMA 1/2)	Mild disease course
SMA025	3a	100	27% (*)	59% (40% CTRL)	Mild disease course
SMA026	3a	100	16% (47% CTRL)	0% (85% CTRL)	Mild disease course
SMA027	3b	0	0% (*)	0% (*)	Mild disease course, ambulatory
SMA028	3b	0	24% (*)	37% (*)	Mild disease course, ambulatory
SMA029	3b	99	0% (80% CTRL)	0% (52% CTRL)	Mild disease course, ambulatory

The healthy control group is not listed since all samples were classified correctly. (*) wrong assignment with CTRL 2 m-19 years only

CTRL, Control; SMA, spinal muscular atrophy; *s/p*, status post

**Fig. 2:**
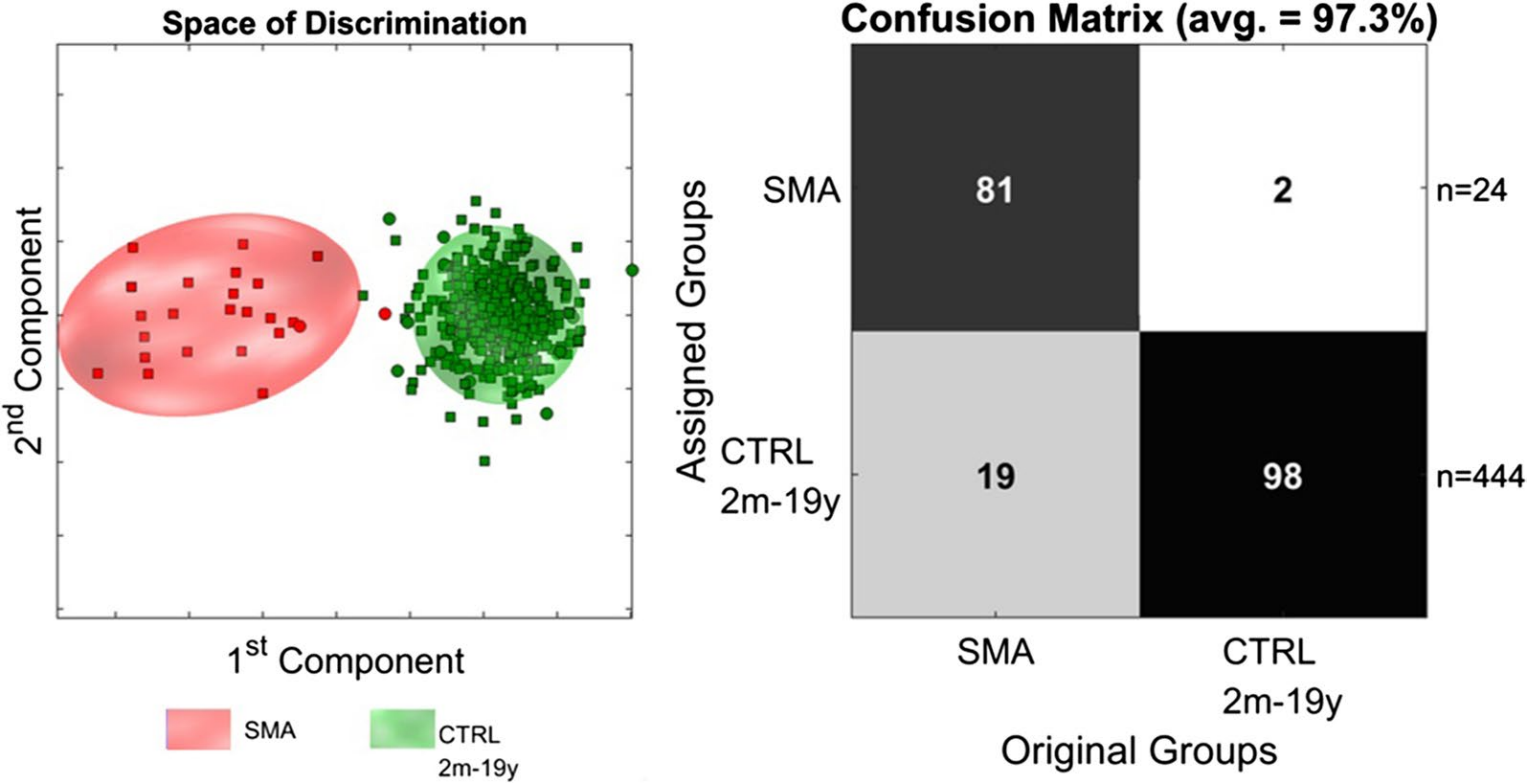
Discrimination between SMA patients and healthy controls. PCA/CA classification and MCCV showed clear discrimination between the SMA group and an age-matched healthy control group. PCA/CA was performed on 1,000 variables from 0.5 to 10 ppm (exclusion: see Materials and Methods) with Expl. Variance of 99.9%. The Confusion Matrix is the result of 100 Monte-Carlo-Runs (MC) with 24-fold CrossValidation (CV). Space of discrimination is one representation of the modelling samples in 2-dimensions. □ represent the model-set and ○ represent the test-set. *SMA:* spinal muscular atrophy. *CTRL:* control

PCA/CA/k-NN classification of urinary metabolic profiles showed discrimination between the SMA and control group with 81% sensitivity and 98% specificity for recognizing SMA (Fig. 2). False assignment occurred in two ambulatory SMA 3b patients (SMA027, SMA028), in one SMA 1 (SMA008), one SMA 2 (SMA020) and one SMA 2/borderline SMA 3 (SMA015) patient, respectively.

**Fig. 3:**
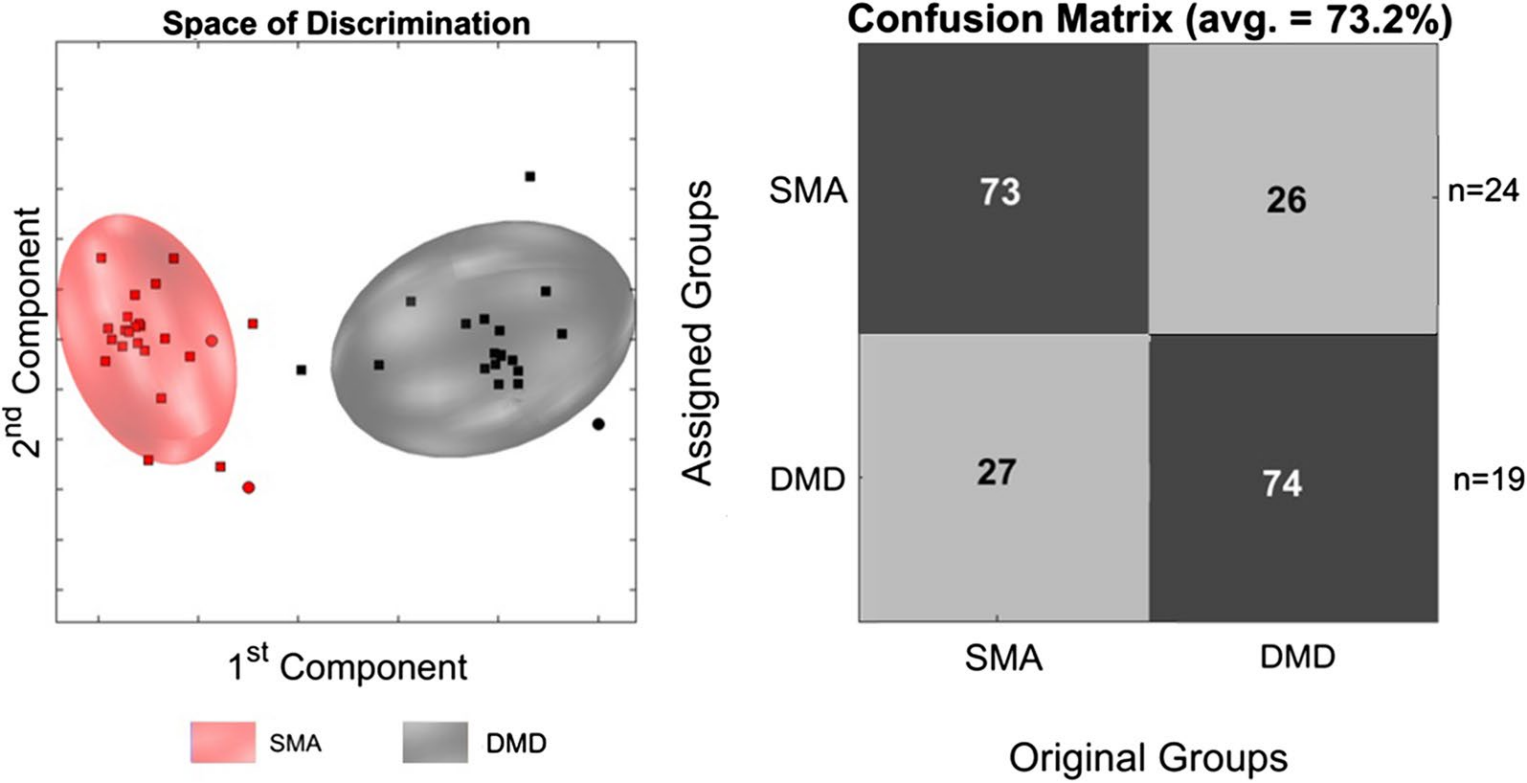
Discrimination between SMA and DMD patients. PCA/CA classification and MCCV showed clear discrimination between the SMA group and the DMD group. PCA/CA was performed on 1,000 variables from 0.5 to 10 ppm (exclusion: see Materials and Methods) with Expl. Variance of 99.9%. The Confusion Matrix is the result of 100 Monte-Carlo-Runs (MC) with 15-fold CrossValidation (CV). Space of discrimination is one representation of the modelling samples in 2-dimensions. □ represent the model-set and ○ represent the test-set. *SMA:* spinal muscular atrophy. *DMD:* Duchenne muscular dystrophy

To exclude an ascertainment bias by unspecific changes in muscle metabolism, we compared the SMA group to another childhood-onset neuromuscular disease, DMD. Twenty-four urinary samples of SMA patients (male-to-female ratio 0.7:1, median age 7.4 (IQR = 13.1) years) were compared to 18 urinary samples of DMD patients (all male, median age 7.5 (IQR = 8.5) years). Metabolic profiles of these two diseases showed spatial discrimination correctly classifying SMA patients with 73% sensitivity and 74% specificity (Fig. 3). Exclusion of female patients from the SMA group to assure equal gender distribution returned comparable results (Additional file 2: Fig. S2).

Thus, urinary metabolic profiling using ^1^H-NMR spectroscopy can provide specific and recognizable spectral fingerprints for SMA.

### Urinary metabolic profiles can help in predicting SMA disease severity

To assess whether urinary metabolic profiles can reflect disease severity and to refine our predictions, we divided the SMA cohort into subgroups based on clinical classification and established two prediction models (Table 2).

**Fig. 4:**
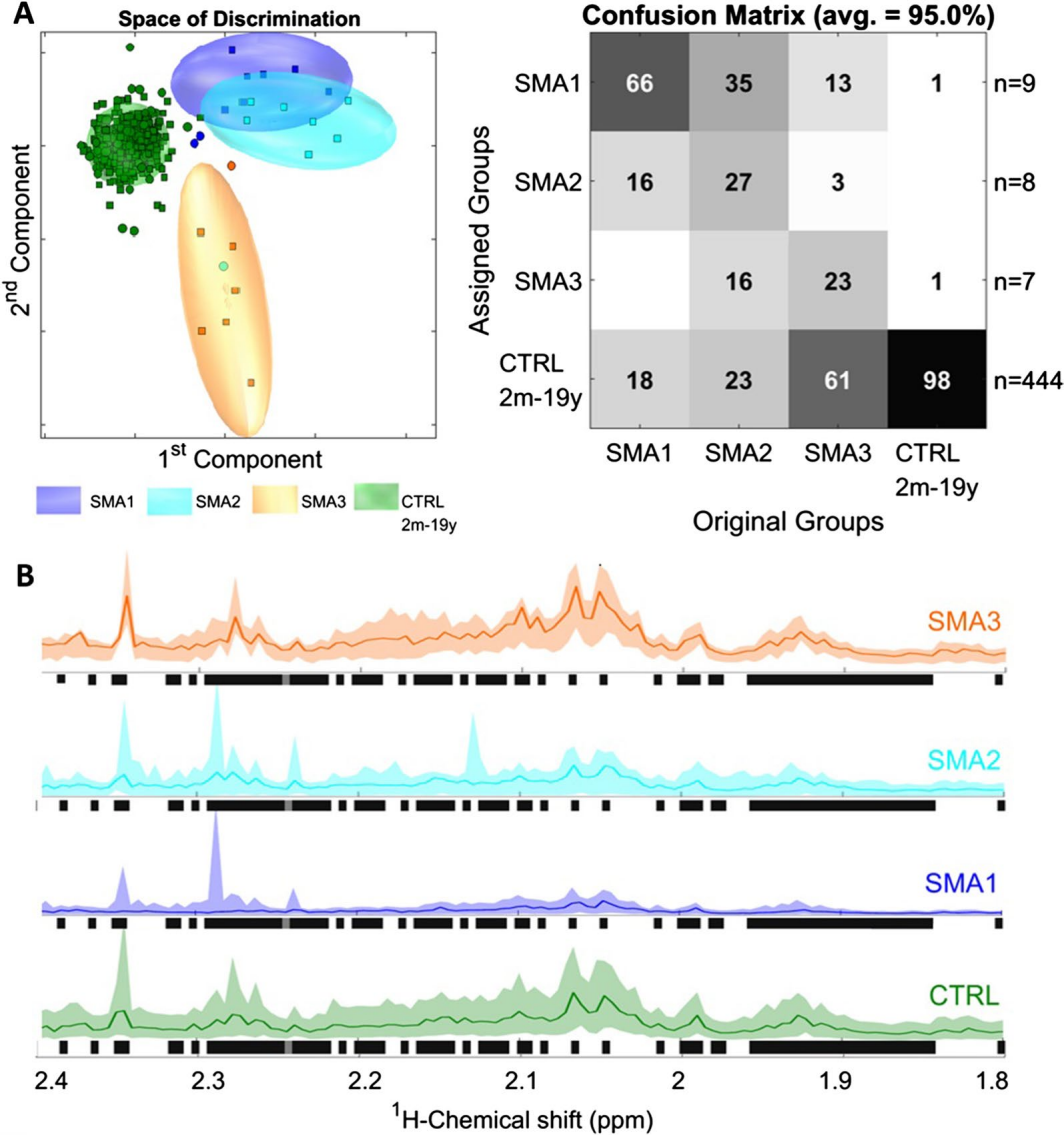
Disease prediction. **A** PCA/CA classification and MCCV of the SMA 1, 2 and 3 group and an age-matched healthy control group showed clear discrimination between the SMA 1 and SMA 3 group with some overlap between the SMA 1 and 2 group as well as the SMA 3 group and controls. PCA/CA was performed on 1,000 variables from 0.5 to 10 ppm (exclusion: see Materials and Methods) with Expl. Variance of 99.9%. The Confusion Matrix is the result of 100 Monte-Carlo-Runs (MC) with sevenfold CrossValidation (CV). Space of discrimination is one representation of the modelling samples in 2-dimensions. **B** Expansion of 6% of overall ^1^H NMR spectra. The Kruskal–Wallis test of ^1^H NMR spectra of the different SMA types and controls revealed significant protein backgrounds in the aliphatic region as one of the major discriminants. Colored lines represent medians and colored areas correspond to variation (12.5–87.5% quantile) of ^1^H-NMR spectra. Spectral regions highlighted in black illustrate significant differences between the 4 groups (*p*-value < 0.01). *SMA:* spinal muscular atrophy. *CTRL:* control

First, three subgroups of children between 2 months and 19 years were selected (9 SMA 1, 8 SMA 2 and 7 SMA 3) and compared to each other and healthy controls (Fig. 4A). Again, controls were correctly classified as healthy in almost all cases (98%). The clinically severely affected SMA 1 subgroup was correctly recognized in 66% of cases. Two patients were falsely assigned to the SMA 2 group (SMA013) and to controls (SMA011), respectively. False assignment of SMA 1 patients to the SMA 3 group did not occur. The SMA 2 group was correctly recognized in 27% of cases with significant overlap to the SMA 1 group (SMA016, SMA019, SMA020 and SMA021). However, despite overlap among one another, the SMA 1 and SMA 2 subgroups showed good discrimination from the SMA 3 subgroup (0% and 16% false assignment, respectively). Inversely, the SMA 3 group demonstrated good discrimination from SMA 1 and 2, false assignment occurred in 13% and 3% of cases, respectively. Along these lines, almost all SMA 3 patients showed a certain degree of overlap with healthy controls with a 61% false negative rate. Closer analysis of ^1^HMR spectra revealed multiple regions of the metabolome being responsible for the detected differences. The most pronounced changes were predominantly driven by protein background, particularly in the severely affected type 1 patients (Fig. 4B) and seemed to dynamically change under therapy with Nusinersen as demonstrated by longitudinal analysis of individual SMA013 (Additional file 3: Fig. S3). The exact proteins involved are still under investigation. Currently, the major discriminants for the changes in protein background could be localized to the aliphatic region of the metabolome, a region containing molecule classes such as alkanes, alkenes, alkynes and their derivates. Some of these key metabolites are depicted in Additional file 3: Fig. S3.

**Fig. 5:**
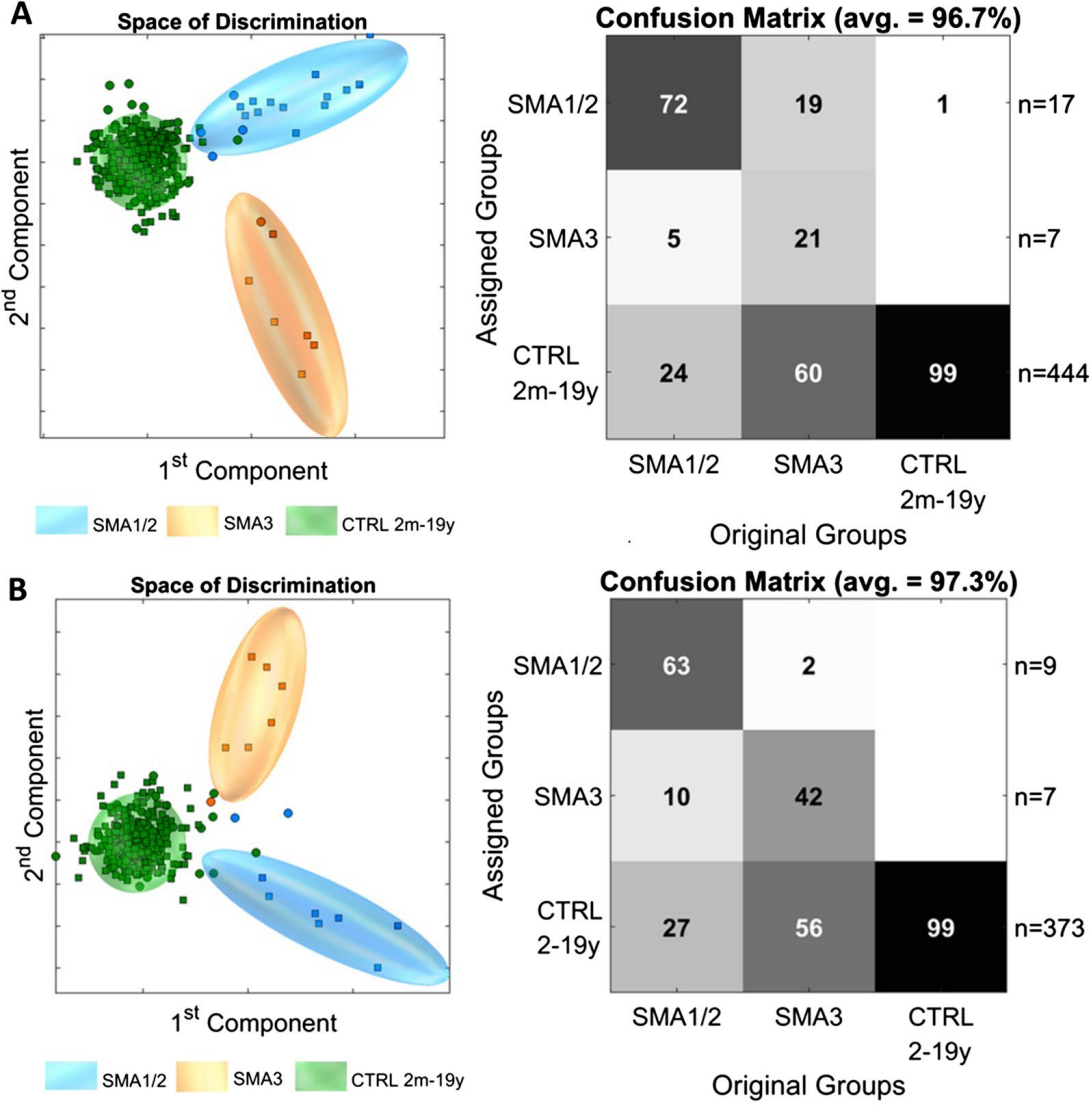
Early vs. late symptomatic SMA. **A** PCA/CA classification and MCCV of early symptomatic (SMA 1/2) vs. late symptomatic (SMA 3) group in relation to an age-matched control group of healthy individuals between 2 months and 19 years showed clear discrimination between early and late symptomatic SMA. **B** Similar results were returned after excluding children under 2 years rendering age bias as confounder unlikely. PCA/CA was performed on 1,000 variables from 0.5 to 10 ppm (exclusion: see Materials and Methods) with Expl. Variance of 99.5%. The Confusion Matrix is the result of 100 Monte-Carlo-Runs (MC) with sixfold CrossValidation (CV). Space of discrimination is one representation of the modelling samples in 2-dimensions. □ represent the model-set and ○ represent the test-set. *SMA:* spinal muscular atrophy. *CTRL:* control

Following these observations, and supported by the results of muscle function tests (Table 1), we next clustered the SMA 1 and 2 subgroups to form an “early symptomatic” group (i.e. children with disease onset before 18 months, who never achieved the ability of independent walking) and compared them to the SMA 3 subgroup as “late symptomatic” group (i.e. children with disease onset between 18 months and 19 years, who achieved independent walking) (Fig. 5A). MCCV showed correct identification of SMA 1/2 in 72% of cases with false assignment to SMA 3 in only 5% of cases. Inversely, only 19% of SMA 3 patients were falsely assigned to the early symptomatic SMA 1/2 group. Importantly, 6 of the 8 SMA 1 and 2 patients that were falsely classified with the previous model were correctly assigned to the respective groups using the clustered “early vs. late symptomatic” approach. By contrast, three clinically clear SMA 1 patients (SMA006, SMA007 and SMA010), who were correctly classified with the previously model, were now assigned to the control group. Again, significant overlap between the SMA 3 group and healthy controls occurred (60%). To reduce age bias, we next excluded children under 2 years of age from the analysis (Fig. 5B). As a result, 63% of the early symptomatic SMA 1/2 group were correctly assigned, while false assignment to the late symptomatic SMA 3 group occurred in 10% of cases. Importantly, only 2% of the SMA 3 patients were falsely assigned to the SMA 1/2 group. Age as confounding factor for the separation between early symptomatic and late symptomatic patients can, therefore, be widely excluded.

To assess, whether the observed changes can dynamically change under therapy, we analyzed longitudinal follow-up samples of one SMA1 patient (SMA013) under Nusinersen treatment suggesting a dose-dependent effect (Additional file 3: Fig. S3).

Thus, both prediction models, although depicting a number of false classifications, were able to assign the majority of SMA patients to the correct phenotypic subgroups, with a particularly good separation between mildly and severely affected patients.

### Urinary metabolic profiles of pre-symptomatic SMA patients can potentially predict disease severity

**Fig. 6:**
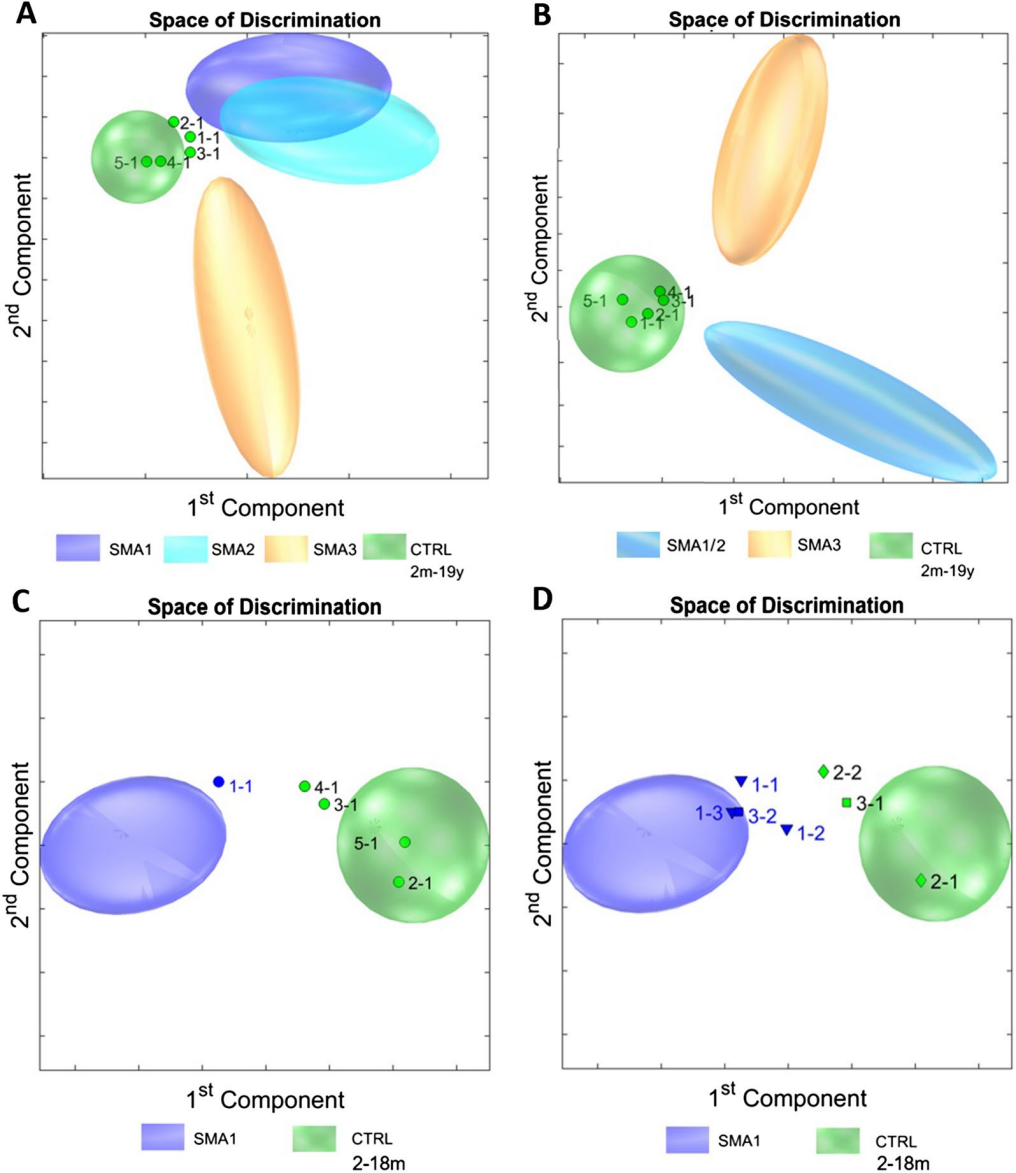
Prediction of disease severity in pre-symptomatic SMA. **A** Spectroscopic fingerprints of five pre-symptomatic SMA samples (determined as 1-1, 2-1, 3-1, 4-1, and 5-1) in comparison to SMA 1, SMA 2, SMA 3 and controls between 2 months and 19 years were consistent with the control group. **B** Comparison of pre-symptomatic SMA vs. early symptomatic SMA (SMA 1/2), late symptomatic SMA (SMA 3) and controls between 2 and 19 years returned similar results. **C** Age-matched comparison with SMA 1 and healthy controls between 2 and 18 months reveals a borderline fingerprint of patient SMA001 (1-1) showing some similarities to the SMA 1 profile. **D** Interestingly, follow-up urine samples from patient SMA001 after 6 months (1-2) and after 8 months (1-3), as well as a follow-up urine sample of patient SMA003 (3-1) after 4 months (3-2) confirm high similarities with the SMA 1 group. No changes were observed on a follow-up urinary sample of patient SMA002 (2-1) after 2.5 months (2-2). *SMA:* spinal muscular atrophy. *CTRL:* control

To address whether metabolic profiles can provide predictions of pre-symptomatically identified SMA patients, we investigated the urinary metabolic profiles of 5 pre-symptomatic children, identified through genetic newborn screening, compared to symptomatic patients and healthy controls (Fig. 6).

PCA/CA/k-NN classification of pre-symptomatic individuals compared to all SMA patients and healthy controls from 2 months to 19 years (Fig. 6A) as well as to early symptomatic SMA 1/2 and late symptomatic SMA 3 patients from 2 to 19 years (Fig. 6B) assigned 100% of the pre-symptomatic group to controls, in agreement with the absence of clinical signs. However, since the pre-symptomatic group consisted of very young children only, we next conducted an age-matched experiment including only pre-symptomatic patients, SMA 1 and controls from 2 to 18 months (Fig. 6C). As a result, one of the pre-symptomatic patients (SMA001) was classified as borderline SMA 1. The changes observed involved subtle alterations in large parts of the metabolic profile with emphasis on protein background. Follow-up urinary profiles of SMA001 after 6 and 8 months of age progressively converged with the SMA 1 group (Fig. 6D). Interestingly, this patient, after being able to sit with 9 months and walk with 13 months, developed signs of mild proximal weakness prompting initiation of Nusinersen treatment with 16 months of age. To a lesser degree, a follow-up urinary profile of SMA003 after 4 months changed towards borderline SMA 1. The patient is currently 27 months old and shows age-expected motor development without treatment. No changes were seen on a follow-up urinary sample of SMA002 after 2.5 months. SMA002 was started on pre-symptomatic Nusinersen treatment with 6 months of age and on last follow-up with 13 months showed age-expected motor development. No follow-up urine samples were available for patients SMA004 and SMA005 who were asymptomatic on last clinical follow-up with 21 and 15 months of age respectively without Nusinersen treatment.

Thus, urinary metabolic profiles of pre-symptomatic SMA patients show mild changes which might announce biochemical onset of disease preceding clinical deterioration.

## Discussion

SMA is a disabling and life-limiting neuromuscular disease. Novel therapies have shown to improve clinical outcomes. Yet, the absence of reliable diagnostic and prognostic biomarkers renders the determination of clinical disease onset challenging, especially after a positive newborn screening result. Current treatment recommendations do not adequately reflect the complex pathogenesis of SMA. This will lead to both delayed treatment initiation in clinically severely affected individuals (e.g. in children with 4 *SMN2* copies), limiting therapeutic effects, as well as unnecessary early administration of costly and invasive, potentially harmful therapies in mildly affected patients. Thus, the need for reliable diagnostic and prognostic biomarkers remains very relevant. Another problem in daily practice is that a large number of newborns with 2 *SMN2* copies and a positive SMA newborn screening result are already symptomatic at time of treatment initiation, exact predictions about their potential motor development are still imprecise. Many studies have investigated the value of electrophysiological, radiologic and laboratory measurements [26–30]. Particularly, plasma phosphorylated neurofilament heavy chain (pNF-H), SMN protein and serum creatinine levels have been proposed as promising biomarkers for disease activity and treatment response in SMA [6, 11, 25, 31, 32]. Indeed, spot urine creatine-to-creatinine quotients differed significantly in our cohort with SMA 1/2 patients showing highly elevated ratios, while SMA 3 patients showed moderately elevated ratios (Fig. 1D). Yet, none of the proposed substrates alone showed sufficient sensitivity and specificity to guide clinical decisions. Considering the complexity of different, partly unknown, modifiers involved in determining SMA phenotypes, the evaluation of the entire metabolome, rather than individual metabolites, might provide a more comprehensive outcome measure for disease severity as foundation for therapeutic decisions.

Metabolic profiling describes the large scale quantitative and qualitative analysis of low molecular weight chemicals (< 1 kDa) present in biological samples [33]. Metabolic profiles can be considered as an organism’s ultimate biochemical output integrating genetic and environmental modifiers, showing high correlation to clinical phenotypes [17]. NMR-based metabolomics offers many advantages in biomarker development. NMR is a high-throughput method requiring minimal sample preparation, allowing quantitative analysis with excellent reproducibility and unlike commonly used techniques, such as mass spectrometry, simultaneously measures all of the more abundant compounds within a biological sample [34, 35]. Urine has been proposed as the preferred medium for biomarker development in neurological diseases including SMA, since it is readily available and easily obtained in a non-invasive manner and more importantly, unlike blood and cerebrospinal fluid, lacks homeostatic mechanisms that might attenuate systemic fluctuations, thus more adequately reflecting disease-specific changes [18, 24]. Along these lines, urinary biomarkers have been identified in a number of CNS diseases, including neuropsychiatric, demyelinating, neurodegenerative and motor neuron diseases [19].

In this study, we conducted a proof-of-concept and feasibility approach using a highly standardized and strictly quality controlled ^1^H-NMR-based metabolic profiling platform along with sophisticated machine learning algorithms to explore the value of urinary metabolic profiles in identifying disease-specific and predictive signatures in urine samples of 29 treatment-naïve SMA patients. Our results suggest that ^1^H-NMR spectra might provide recognizable metabolic fingerprints for SMA, allowing discrimination from healthy controls and the neuromuscular disease DMD. Importantly, profiles of affected SMA patients, in the majority of cases, displayed some correlation to phenotypic severity and might be able to reflect dynamic changes under therapy. Close analysis of metabolic spectra revealed multiple regions of the metabolome (most importantly protein background and in particular the aliphatic region) being responsible for the separation between SMA subgroups and controls, emphasizing the concept of complex and multifactorial biochemical changes driving SMA phenotypes, as opposed to a single-biomarker based approach to predict disease severity. The exact proteins involved are still under investigation.

Based on our findings, two prediction models were established, with moderate sensitivity. Yet, sensitivity proved to be good in separating the extreme ends of the spectrum (early-onset SMA 1 vs. late SMA 3 with relatively long preserved ambulation). A few patients were assigned to the wrong groups. For instance, SMA015, a one-year-old girl with mild SMA 2/borderline SMA 3, who on clinical follow-up under Nusinersen treatment is currently able to stand and walk first steps, was constantly assigned to the healthy control group, potentially reflecting residual motor capacity after relatively early initiation of therapy. By contrast, SMA018, a severely affected SMA 2 patient with mild intellectual disability and multiple comorbidities, including pulmonary hypertension due to Ebstein anomaly and epileptic seizures requiring treatment with sildenafil and valproic acid, respectively, was classified as SMA 3. We are currently unable to confirm whether these comorbidities or co-medications caused false classification. Further, significant overlap between the SMA 3 group and controls occurred (SMA024, SMA026, SMN027, SMA028 and SM029). This observation was likely caused by mild disease, including partly preserved ability to walk. Motor performance/capacity on time of sample collection seemed to play a role for biochemical classification, since patients with mild phenotypes (SMA 3, particularly with preserved ambulation) were recognized as healthy in the majority of cases, while SMA 2 patients with ongoing alpha motor neuron degeneration for many years, showed some overlap with the SMA 1 group (SMA019, SMA020, SMA021). These results support the notion of a more nuanced classification of the SMA disease spectrum along a phenotypic continuum, rather than the rigid traditional division into four clinical phenotypes, as has in a similar fashion been proposed for other neuromuscular diseases [36].

Interestingly, age-matched analysis of pre-symptomatic SMA children, identified by genetic newborn screening, revealed heterogeneous results with most patients being assigned to the control group, as expected due to the absence of clinical signs, however classifying one patient as borderline SMA 1. Follow-up urinary metabolic signatures of this patient with 6 and 8 months progressively converged with the SMA 1 group, preceding signs of mild proximal muscle weakness becoming apparent after 13 months of age. A second patient, showing very mild changes of the metabolic signature drifting towards borderline SMA 1 is currently developing normally.

Our results demonstrate that ^1^H-NMR-based metabolic profiling of urinary samples is feasible and might hold the potential to complement current treatment algorithms in SMA, providing an additional tool for diagnosis, disease prediction, therapeutic decision-making, and follow-up under treatment. Clinical applications of this method might be broad, if proven to be reliable in larger studies. Despite promising newborn screening programs all over the world, accurate and early predictions of disease severity remain a challenging issue. Our results suggest that urinary metabolic profiling of pre-symptomatic children might depict biochemical onset of disease before clinical signs become visible, providing potentially quantifiable measurements for clinical decisions and prognosis with respect to disease onset and initiation of specific therapy and opening new avenues in patient stratification. Moreover, since preliminary results suggest that urinary signatures dynamically change under Nusinersen therapy, the value of ^1^H-NMR based metabolomics as therapeutic monitoring tool might be worth further exploring in follow-up studies. Comparing different therapies and quantifying therapeutic effects, especially given the multiple existing treatment regimens for SMA, including Nusinersen, Risdiplam and gene therapy, will be an important future task and could be of relevance for evaluating treatment response, comparing therapies with respect to their long-term efficacy, additive effects and potential side effects and might thus help to develop personalized therapeutic concepts. Along these lines, integrating changes of the metabolome under therapy with motor function scores and additional outcome measures might additionally identify individual profiles for responders and non-responders for specific therapies. The baseline metabolic profiles provided in this study might help as comparator in future clinical trials. Finally, the identification of the exact metabolites responsible for the disease-specific pathophysiological fingerprints, through future research, possibly by combining different metabolomic technologies including further high-resolution techniques such as mass spectrometry, could potentially allow targeted analysis of the responsible metabolites with diagnostic metabolic panels.

This study depicts several limitations. The small number of treatment-naïve SMA patients enrolled in this study limits the significance of our findings, both in the symptomatic and the pre-symptomatic group which is in great parts attributed to the fact that most patients are nowadays under therapy. Both prediction models proposed in this study, despite showing good sensitivity in separating the mildly from the severely affected patients, lacked sufficient sensitivity in correctly classifying SMA 2 patients and discriminating mildly affected patients from controls. Additional (potentially more accurate) prediction models, for instance classifying patients by motor function at time of sample collection (“non-sitter” vs. “sitter” vs. “walker”), were not feasible due to the small sample size within the subgroups. Due to these limitations, our preliminary results, although encouraging, have to be interpreted with caution and need confirmation in larger studies. Furthermore, potential sources for bias were the age differences between SMA subgroups, which seemed to dominate the discrimination between subgroup-specific spectral fingerprints rendering direct comparison of the different SMA types difficult (Fig. 1B and Additional file 1: Fig. S1). Age-bias was reduced as far as possible by conducting different analyses with age-matched subgroups. Despite showing that age-matched spectral profiles of SMA patients differed from DMD as another neuromuscular disease, we acknowledge that DMD, an X-linked muscular dystrophy only affecting males, might not be the ideal choice since it differs from SMA in terms of pathophysiology and mode of inheritance. We currently cannot confirm whether metabolic profiles can reliably differentiate SMA from clinically important differential diagnoses, such as other motor neuron diseases, congenital myopathies, congenital muscular dystrophies, birth trauma or structural malformations of the CNS.

In order to refine the prediction models proposed in this study and to develop additional and potentially more accurate prediction models, further studies including longitudinal samples of newly diagnosed SMA patients before and under therapy as well as newborn children with SMA, preferably pre-symptomatic individuals identified by newborn screening, along with a broad range of differential diagnoses will be needed. Continuous feeding of the database with additional samples will train the machine learning algorithms and render prediction models increasingly robust and diagnostically conclusive. Standardized collection, preparation and analysis of metabolic spectra and correlation of longitudinal changes in metabolic profiles to clinical performance and to currently proposed biomarkers such as pNF-H, serum creatinine and SMN protein will further validate the potential of this method and might help quantifying clinical effects in study settings and clinical practice.

In conclusion, with this study we carefully explored the value of ^1^H-NMR-based urinary metabolic profiling in biomarker development for SMA. By delineating possible confounders and discussing potential applications and limitations we believe that this proof-of-concept and hypothesis generating study justifies subsequent clinical trials, preferably in a multicenter national or international setting, to evaluate the use of our preliminary findings in clinical practice. Ultimately, and if proven to hold true in future clinical studies with larger patient cohorts involving a sufficiently high number of pre-symptomatic children with SMA, ^1^H-NMR-based urinary metabolic profiling in combination with sophisticated machine learning based prediction algorithms could potentially provide a valuable prognostic tool and outcome measure serving as objective quantifier in comparative therapeutic trials and provide an additional non-invasive method for diagnosis, disease prediction, and therapeutic monitoring, hence aiding in clinical decision-making and design of personalized therapies in the rapidly evolving field of SMA.

## Methods

### Study design and protocol

In this multicenter observational study urine samples were collected from 29 treatment-naïve pediatric and adolescent patients with genetically proven 5q SMA from three German neuromuscular centers, 18 children and adolescents with DMD from Heidelberg University Hospital and 444 healthy pediatric and adolescent controls from two pediatric practices in Reutlingen and Mannheim, Germany. We additionally collected three follow-up urinary samples form one patient under Nusinersen therapy. The clinical protocol was conducted in accordance with the current versions of the Declaration of Helsinki and the Medical Association’s professional code of conduct in Baden-Württemberg, Germany, and was approved by the ethics committees of the Universities of Heidelberg (S-554/2018), Hamburg (MC-265/19) and Munich (18-269) for sample collection of SMA and DMD patients, and the ethics committee of the State Medical Council of Baden Württemberg for sample collection of healthy controls (F-2013-006#A). All patients and their legal guardians enrolled in this study provided signed informed consent.

Since the study started approximately one year after approval of Nusinersen in Europe, newly diagnosed SMA patients before specific treatment could only be recruited consecutively. Patient characteristics of the SMA cohort are summarized in Table 1. SMA type was clinically determined based on age at symptom onset and achievement of highest motor milestones [1]. *SMN2* copy numbers were determined by Multiplex Ligation-dependent Probe Amplification (MLPA) analysis in accredited quality-controlled laboratories. Detailed medical histories and comprehensive neurologic examinations were taken by experienced child neurologists and neurologists with special expertise in the field of SMA. Motor function tests including the Children’s Hospital of Philadelphia Infant Test of Neuromuscular Disorders (CHOP-INTEND), an established motor function test for SMA patients with very limited motor abilities [37], the Hammersmith Functional Motor Scale Expanded (HFSME), a commonly used motor function test for non-ambulant SMA patients [38] and the Revised Upper Limb Module (RULM), a standardized test for the assessment of upper limb performance [39], were conducted by experienced physiotherapists. Since the CHOP-INTEND score shows a "ceiling effect" in the attainment of motor functions, notably a maximum score of 64 points corresponding to the ability of turning from supine to prone position, HFMSE and RULM scores were collected from those children who achieved a higher CHOP INTEND score than 60/64. Therefore, alternatively HFMSE and RULM scores were collected from children above 2 years of age who were able to sit and from children with a CHOP INTEND score ≥ 60/64 points.

### Sample collection

After comparing urine, serum and cerebrospinal fluid (CSF), and since a robust pediatric control cohort for urine already existed, urine was chosen as preferred biofluid for this study (Additional file 4: Fig. S4), in line with previous reports [18, 24]. A single treatment-naïve urine sample was collected from each participant, usually as a fasting morning urine, unless not possible due to very young age and inability of prolonged fasting periods. Effects of diet or medication e.g. anesthetics such as N-acetyltyrosine in case of severely affected patients were identified in the spectra and the corresponding regions were excluded for statistical analysis. Sample collection, sample preparation and NMR analysis were conducted according to published protocols and following strict quality control criteria, such as limitation of freeze–thaw cycles, ring tests, etc. [40–42]. Additionally, random samples were measured multiple times (up to 50 times on different time points during a one-year period) to ensure robustness and reproducibility of the method (Additional file 5: Fig. S5). Samples were processed and stored to the NCT Liquidbank Heidelberg, a part of a local network of quality-ensured biobanks, according to approved standards. Samples were centrifuged at 2500 × *g*_*max*_ for 5 min at 4 °C. The supernatant was aliquoted into 2 mL cryovial each and stored at − 80 °C prior to measurement.

### Sample preparation

Urine samples were prepared according to standard procedures as previously described [43]. Frozen urine samples were thawed at 4 °C and shaken before use. 0.9 mL of urine was added to 0.1 mL of potassium phosphate buffer (pH 7.4) containing trimethylsilylpropionic acid-d_4_ sodium salt (TSP) and sodium azide. The mixture was homogenized, and 0.6 mL were transferred to a 5 mm NMR tube for analysis and placed in a cooled sample changer for analysis.

### ^1^H-NMR spectroscopy analysis

Samples were analyzed in full automation according to standard procedures on a Bruker IVDr System, as previously described [43] using a 600 MHz Bruker Avance III HD NMR spectrometer equipped with an automated sample changer SampleJet with sample cooling and pre-heating station, 5 mm inverse probe with z-gradient and automated tuning and matching and cooling unit BCU-I. TopSpin 3.6 in combination with Bruker’s body fluid NMR methods package B.I. Methods 1.0 was used for fully automated acquisition and processing controlled by ICON NMR.

Prior to measurement, samples were kept for 5 min inside the NMR probehead for temperature equilibration at 27 °C. Tuning and matching, locking, shimming, optimization of the lock phase and calibration of the hard pulse at 90 °C were done automatically for optimization of the NMR experimental conditions. Next, one-dimensional ^1^H-NMR spectra were acquired applying a standard pulse sequence with suppression of the water peak (Bruker pulse program library noesygppr1d). Fourier transformation and fully automated phasing and baseline correction was done via the Bruker standard automation program APK0.NOE. Spectra were quantitatively calibrated via the PULCON principle [44].

The standardized NMR metabolic profiling platform (Avance IVDr system), including SOPs for body fluid sample preparation, NMR measurements and spectra analysis is available from Bruker BioSpin GmbH. The machine learning methodology applied is described by Assfalg et al*.* [45] and Bernini et al*.* [46]. Uniform spectrometer specifications ensured reproducibility of spectral fingerprints allowing researchers who work with an identical NMR metabolic profiling platform and follow the provided SOPs to obtain the same results.

### Statistical analysis

Data analysis was done with MatLab R2018b using in-house developed methods. Distributions of age and motor function scores (CHOP-INTEND, HFMSE, RULM) were reported as median and interquartile range (IQR). Analysis of metabolic spectra was conducted with age-matched subgroups. Significance levels were reported using the Kruskal–Wallis Anova test, *P* values ≤ 0.05 were considered significant.

#### Spectral binning

Prior to any further postprocessing, spectral intensity was scaled to a mmol/L concentration scale. Then, each spectrum was segmented from 0.4 to 10 ppm into consecutive bins of fixed size, 0.0096 ppm, and the pertaining regional integrals (bin intensities) excluding the following regions: (0.840–0.920) ppm, (0.990–1.090) ppm, (1.160–1.220) ppm, (1.375–1.542) ppm, (2.560–2.650) ppm, (2.772–2.900) ppm, (3.030–3.060) ppm, (3.390–3.913) ppm, (3.920–3.950) ppm, (4.040–4.080) ppm, (4.150–4.260) ppm, (4.335–5.680) ppm. Exclusion was necessary to avoid influences from irrelevant variability, i.e. residual water intensity, medication-related metabolites, and to exclude the influence of creatine metabolism which is known to be impaired in SMA but non-specific [25]. As result of the spectral binning procedure a so-called bucket table was generated where columns represented bin numbers and rows represented NMR sample numbers.

#### Principal component analysis (PCA)

PCA is a standard unsupervised multivariate technique performing a coordinate transformation on an initial table in order to try to separate relevant from residual ones, e.g. noise. It ideally projects correlated variance distributed over several variables onto single new variables, the Principal Components, thus simplifying visualization and interpretation. In the context of this study, PCA was used for visualization and as a dimension reduction technique for preparation of data tables for further multivariate statistical analyses.

#### PCA/CA/k-NN classification

A classification approach different from soft independent modelling by class analogy (SIMCA) is needed if a sample needs to be classified with respect to multiple co-existing classes. Starting from a bucket table of a model set of samples, PCA is applied for dimension reduction first. Then, canonical analysis (CA) in combination with MANOVA is applied to determine the subspace for maximum class separation and its respective dimension. Finally, a classification rule is introduced, e.g. via the k-nearest neighbor (k-NN) concept. This gives the PCA/CA/k-NN classification procedure: For classification of a new test sample, it is projected into the PCA-CA subspace first and k-NN is used to assign its class membership.

#### Monte-Carlo embedded cross-validation (MCCV)

PCA/CA/k-NN classification is a supervised method. Related models are established in a supervised manner, where class membership is known for each object during the training phase. In order not to overfit any data, extensive validation is needed. In the context of this study, the MCCV approach has been taken and the objective of the modeling procedure is to maximize the rate of correct classification by optimizing related model parameters. In PCA/CA/k-NN one needs to optimize for example the explained variance of the selected PCA subspace or the segmentation scheme used for sub-model generation as output of the MCCV.

The result of the MCCV provides a so-called confusion matrix with n × n fields (n equals the number of classes to be discriminated), where diagonal fields represent the probability of true classification and off-diagonal fields relate to probabilities of misclassification, e.g. samples of a class A being falsely assigned to a different class B. PCA/CA/k-NN classification and MCCV are further described by Assfalg et al. [45] and Bernini et al. [46].

## Supplementary Information

**Additional file 1: Fig. S1.** Age Differences in healthy controls. PCA/CA classification and MCCV showed clear discrimination between different age subgroups within the healthy control group. Since age was dramatically affecting spectroscopic fingerprints we conducted all experiments with age-matched healthy controls. PCA/CA was performed on 1000 variables from 0.5 to 10 ppm (exclusion: see Materials and Methods) with Expl. Variance of 99.9%. The Confusion Matrix is the result of 100 Monte-Carlo-Runs (MC) with 30-fold CrossValidation (CV). Space of discrimination is one representation of the modelling samples in 2-dimensions. *CTRL<2m*: control aged < 2 months, *CTRL2-12m*: control aged between 2 and 12 months, *CTRL1-19y*: control aged between 1 and 19 years.

**Additional file 2: Fig. S2.** Discrimination between sex-matched SMA and DMD patients. PCA/CA classification and MCCV showed discrimination between age- and sex-matched SMA and DMD patients. PCA/CA was performed on 1000 variables from 0.5 to 10 ppm (exclusion: see Materials and Methods) with Expl. Variance of 99.9%. The Confusion Matrix is the result of 100 Monte-Carlo-Runs (MC) with 75-fold CrossValidation (CV). Space of discrimination is one representation of the modelling samples in 2-dimensions. *DMD*: Duchenne muscular dystrophy,* SMA*: spinal muscular atrophy.

**Additional file 3: Fig. S3.** Major changes located in the aliphatic region and longitudinal analysis of one SMA1 patient. ^1^H-NMR urinary spectra of patient SMA013 (SMA 1, 16y 10m, female) compared to age-matched healthy controls, depict a strong protein background and an elevation of a number of small molecules (e.g. glutamine, 3-Hydroxybutyric acid) in the aliphatic region driving the observed difference between SMA and healthy cohorts. Interestingly, the ^1^H-NMR metabolic fingerprint of patient SMA013 dynamically evolved during Nusinersen therapy reaching a signature comparable to the healthy control cohort after the 3rd Nusinersen injection. The grey area corresponds to ^1^H-NMR spectra variation (12.5%–87.5% quantile) in the healthy control cohort (CTRL 2m-19y, n = 444). The 4 different colored lines represent *1*H-NMR spectra from the same patient (SMA013) at 4 different time points before (black line: treatment naïve) under therapy with Nusinersen (blue line: before 2nd Nusinersen injection, red line: before 3rd Nusinersen injection, green line: before 4th Nusinersen injection). *CTRL*: control, *T*: timepoint.

**Additional file 4: Fig. S4.** Standardized ^1^H-NMR spectra of urine, plasma and CSF. Overview of standardized ^1^H-NMR spectra of urine, plasma and CSF obtained for all SMA patients included in the study. Colored lines in each plot represent ^1^H-NMR spectra from different patients (blue: SMA 1, cyan: SMA 2, orange: SMA 3). Urine appeared to be the most complex and most informative biofluids with more than 1000 visible compounds. *SMA*: spinal muscular atrophy.

**Additional file 5: Fig. S5.** Long-term reproducibility of urinary NMR spectra. Quality Control (QC) urine samples have been prepared using the same protocols as the patient samples and measured at different time points during the entire study period (over 1 year) in order to monitor the short- and long-term reproducibility of the complete NMR based urinary metabolic profiling workflow. 2 QC urine samples have been prepared, measured and analyzed prior to measuring SMA urine samples. In total, 50 QC urine samples have been measured and the concentrations of different endogeneous metabolites have been determined automatically. Root mean square error (RMSE) and coefficient of variation (CV) showed excellent reproducibility of ^1^H-NMR spectra allowing to detect subtle changes in the disease course.

## Data Availability

The data that support the findings of this study are available on request from the corresponding author, [AZ]. The data are not publicly available due to their containing information that could compromise the privacy of study participants.

## Abbreviations

^1^H-NMR: ^1^H-nuclear magnetic resonance
CHOP-INTEND: Children’s Hospital of Philadelphia Infant Test of Neuromuscular Disorders
CNS: Central nervous system
CSF: Cerebrospinal fluid
DMD: Duchenne muscular dystrophy
HFSME: Hammersmith functional motor scale expanded
MLPA: Multiplex ligation-dependent probe amplification
RULM: Revised upper limb module
SMA: Spinal muscular atrophy
SMN: Survival motor neuron
