# Supplementary figures and images for: 1H-NMR-based metabolic profiling identifies non-invasive diagnostic and predictive urinary fingerprints in 5q spinal muscular atrophy
Source: Orphanet J Rare Dis. 2021 Oct 20;16:441. doi: 10.1186/s13023-021-02075-x (PMC8527822; doi:10.1186/s13023-021-02075-x)

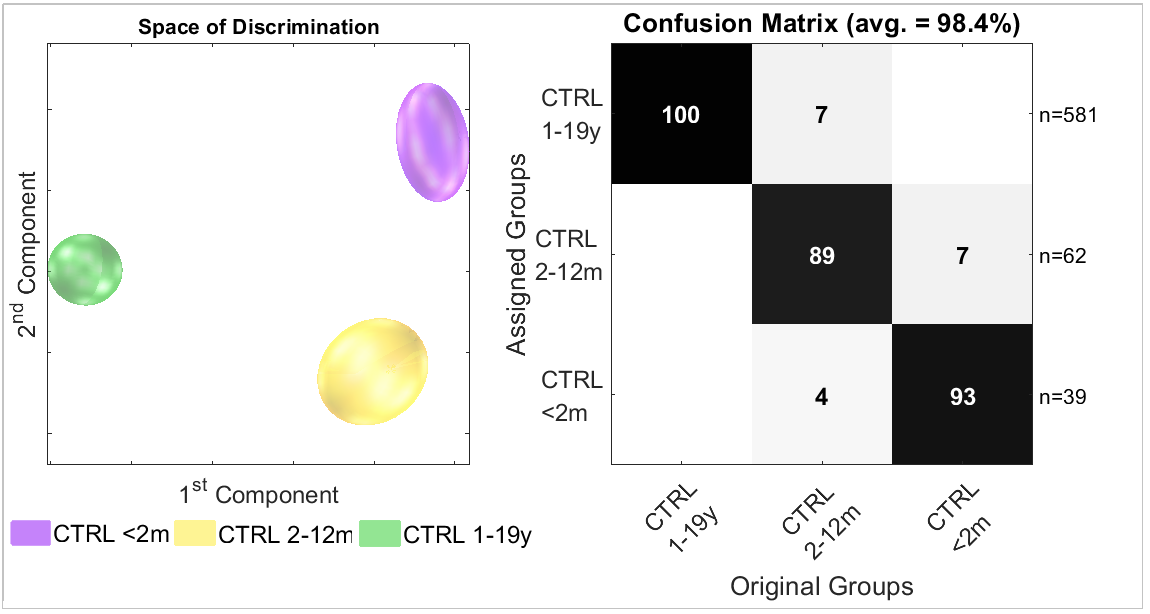

Supplement: Supplementary file 1 — Additional file 1: Fig. S1. Age Differences in healthy controls. PCA/CA classification and MCCV showed clear discrimination between different age subgroups within the healthy control group. Since age was dramatically affecting spectroscopic fingerprints we conducted all experiments with age-matched healthy controls. PCA/CA was performed on 1000 variables from 0.5 to 10 ppm (exclusion: see Materials and Methods) with Expl. Variance of 99.9%. The Confusion Matrix is the result of 100 Monte-Carlo-Runs (MC) with 30-fold CrossValidation (CV). Space of discrimination is one representation of the modelling samples in 2-dimensions. CTRL<2m: control aged < 2 months, CTRL2-12m: control aged between 2 and 12 months, CTRL1-19y: control aged between 1 and 19 years. [file 13023_2021_2075_MOESM1_ESM.tiff]

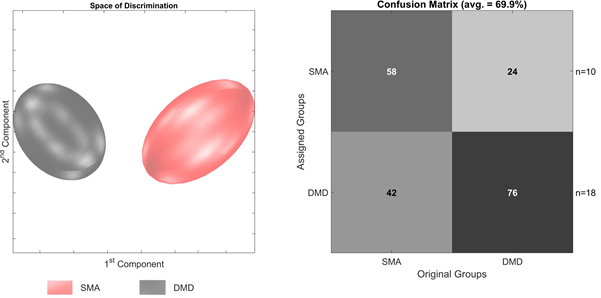

Supplement: Supplementary file 2 — Additional file 2: Fig. S2. Discrimination between sex-matched SMA and DMD patients. PCA/CA classification and MCCV showed discrimination between age- and sex-matched SMA and DMD patients. PCA/CA was performed on 1000 variables from 0.5 to 10 ppm (exclusion: see Materials and Methods) with Expl. Variance of 99.9%. The Confusion Matrix is the result of 100 Monte-Carlo-Runs (MC) with 75-fold CrossValidation (CV). Space of discrimination is one representation of the modelling samples in 2-dimensions. DMD: Duchenne muscular dystrophy, SMA: spinal muscular atrophy. [file 13023_2021_2075_MOESM2_ESM.tiff]

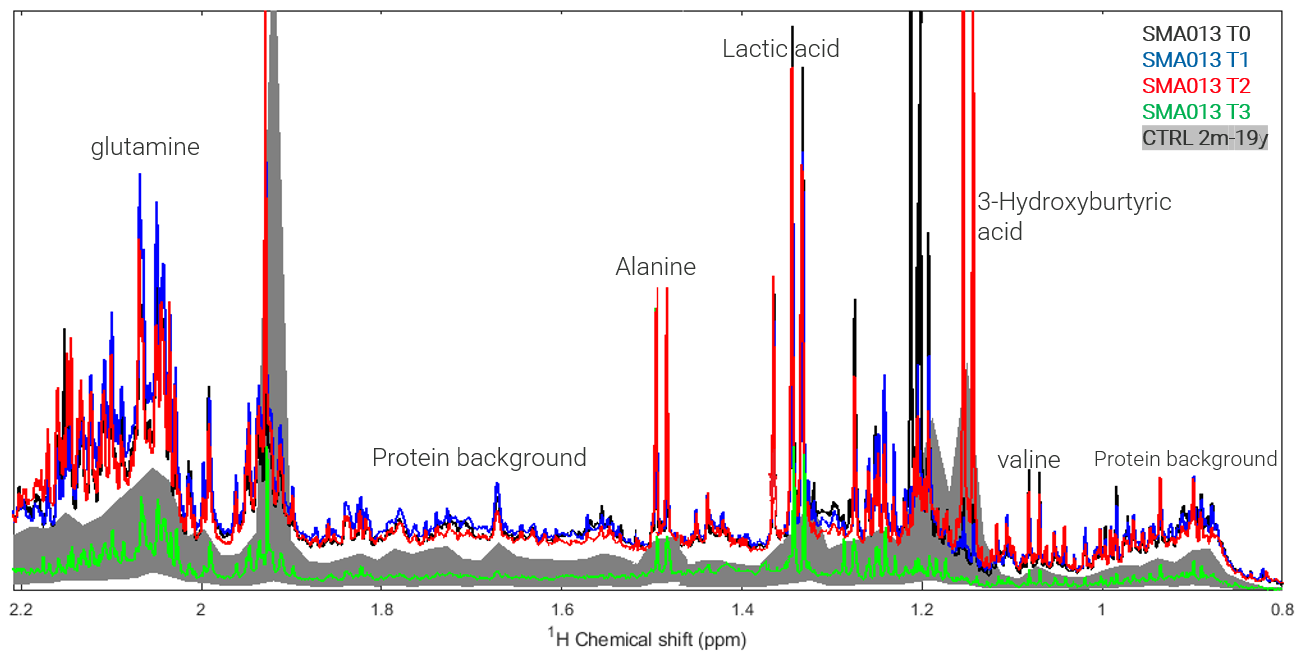

Supplement: Supplementary file 3 — Additional file 3: Fig. S3. Major changes located in the aliphatic region and longitudinal analysis of one SMA1 patient. 1H-NMR urinary spectra of patient SMA013 (SMA 1, 16y 10m, female) compared to age-matched healthy controls, depict a strong protein background and an elevation of a number of small molecules (e.g. glutamine, 3-Hydroxybutyric acid) in the aliphatic region driving the observed difference between SMA and healthy cohorts. Interestingly, the 1H-NMR metabolic fingerprint of patient SMA013 dynamically evolved during Nusinersen therapy reaching a signature comparable to the healthy control cohort after the 3rd Nusinersen injection. The grey area corresponds to 1H-NMR spectra variation (12.5%–87.5% quantile) in the healthy control cohort (CTRL 2m-19y, n = 444). The 4 different colored lines represent 1H-NMR spectra from the same patient (SMA013) at 4 different time points before (black line: treatment naïve) under therapy with Nusinersen (blue line: before 2nd Nusinersen injection, red line: before 3rd Nusinersen injection, green line: before 4th Nusinersen injection). CTRL: control, T: timepoint. [file 13023_2021_2075_MOESM3_ESM.tiff]

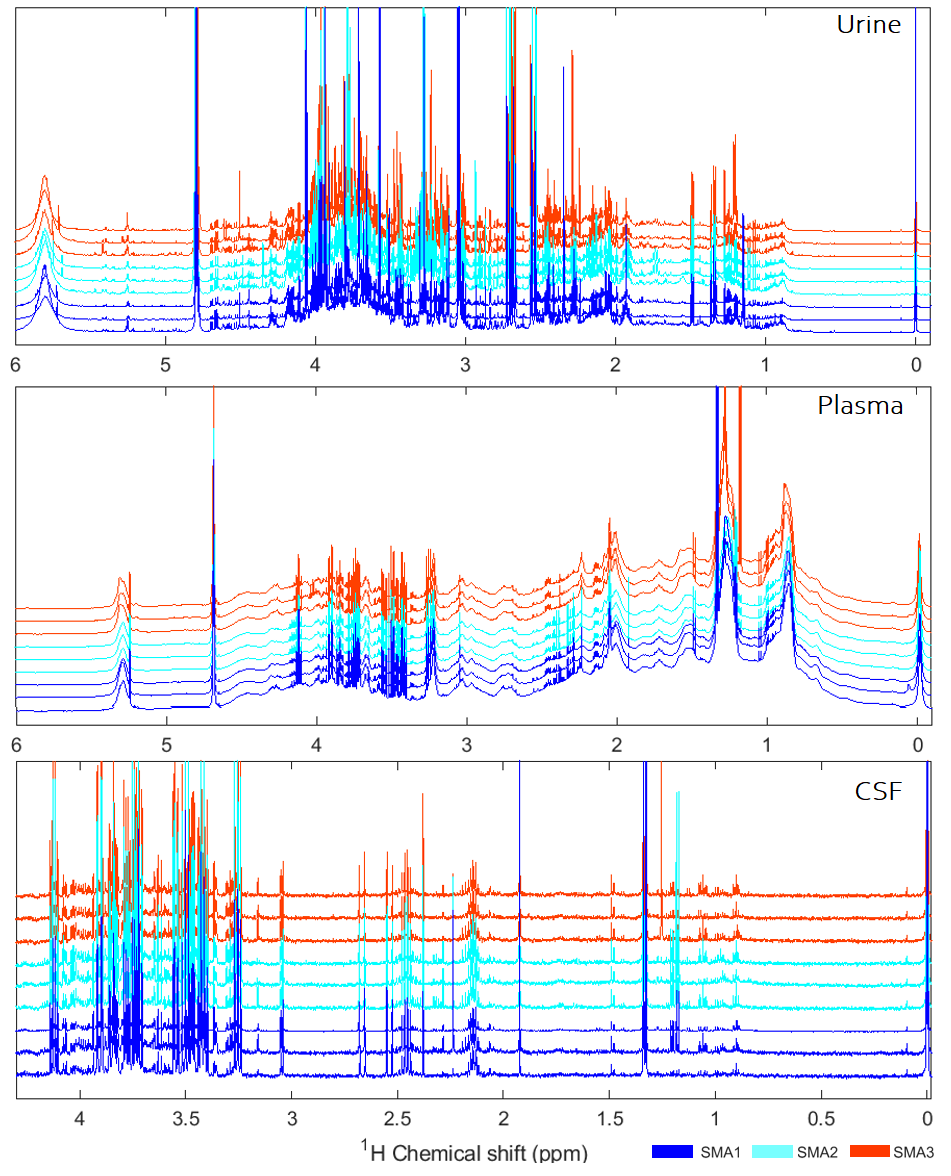

Supplement: Supplementary file 4 — Additional file 4: Fig. S4. Standardized 1H-NMR spectra of urine, plasma and CSF. Overview of standardized 1H-NMR spectra of urine, plasma and CSF obtained for all SMA patients included in the study. Colored lines in each plot represent 1H-NMR spectra from different patients (blue: SMA 1, cyan: SMA 2, orange: SMA 3). Urine appeared to be the most complex and most informative biofluids with more than 1000 visible compounds. SMA: spinal muscular atrophy. [file 13023_2021_2075_MOESM4_ESM.tiff]

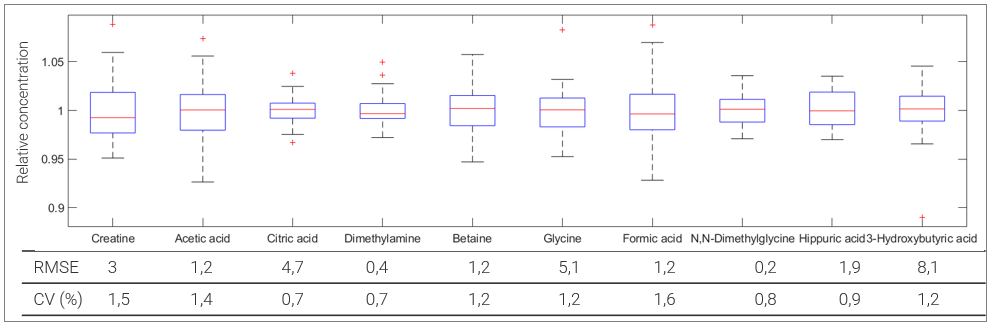

Supplement: Supplementary file 5 — Additional file 5: Fig. S5. Long-term reproducibility of urinary NMR spectra. Quality Control (QC) urine samples have been prepared using the same protocols as the patient samples and measured at different time points during the entire study period (over 1 year) in order to monitor the short- and long-term reproducibility of the complete NMR based urinary metabolic profiling workflow. 2 QC urine samples have been prepared, measured and analyzed prior to measuring SMA urine samples. In total, 50 QC urine samples have been measured and the concentrations of different endogeneous metabolites have been determined automatically. Root mean square error (RMSE) and coefficient of variation (CV) showed excellent reproducibility of 1H-NMR spectra allowing to detect subtle changes in the disease course. [file 13023_2021_2075_MOESM5_ESM.tiff]
